# Implication of Socio-Demographics on Cognitive-Related Symptoms in Sports Concussion Among Children

**DOI:** 10.1186/s40798-016-0058-8

**Published:** 2016-09-14

**Authors:** Laurens Holmes, Joshua Tworig, Joseph Casini, Isabel Morgan, Kathleen O’Brien, Patricia Oceanic, Kirk Dabney

**Affiliations:** 1 Nemours/Alfred I. DuPont Hospital for Children, Office of Health Equity and Inclusion, P.O. Box 269, Wilmington, DE 19803 USA; 2 University of Delaware, Newark, DE 19716 USA; 3 Villanova University, Villanova, PA 19085 USA; 4 Johns Hopkins Bloomberg School of Public Health, Baltimore, MD 21205 USA; 5 Orthopedic Department, Nemours/A. I. duPont Hospital for Children, Wilmington, DE 19803 USA

## Abstract

**Background:**

Sports-related concussion remains a public health challenge due to its morbidity and mortality. One of the consequences of concussion is cognitive impairment (CI) and cognitive-related symptoms (CRS) which determine, to some extent, physical and behavioral functioning of children who sustain concussion. Despite the high prevalence of CI and CRS associated with concussion, the risk factors are not fully understood. We aimed to characterize CRS and to examine its relationship with race, ethnicity, age, insurance, and sex in a pediatric population.

**Methods:**

A retrospective cohort (case-only) design was used to assess CRS prevalence and its relationship with race and sex using a pediatric hospital’s electronic medical records. A consecutive sample was used with 1429 cases between 2007 and 2014. Study characteristics were examined using chi-square and log binomial regression for hypothesis-specific testing.

**Results:**

Of the 1429 cases, 872 (61.0 %) were boys and 557 (39.0 %) were girls. The racial distribution indicated 1146 (80.2 %) Whites, 170 (11.9 %) Blacks/African Americans, and 113 (7.9 %) others. The prevalence of CRS was 78.0 %. Whereas boys had sustained more concussions, girls were more likely to present with CRS; prevalence risk ratio = 1.07, 95 % CI 1.01–1.13, *p* = 0.02. The crude analysis indicated no racial disparities in CRS prevalence, but the multivariable analysis did, comparing White to Black/African American children; adjusted prevalence risk ratio (aPRR) = 1.77, 99 % CI 1.02–3.08, *p* = 0.008.

**Conclusions:**

Racial disparities exist in CRS among children with sports-related concussion, and Black/African American children are more likely, relative to Whites, to suffer CRS. Due to uncertainty in causal inference, we caution the interpretation and application of these data in risk-adapted concussion prevention.

## Key Points

- Cognitive-related symptoms (CRS) are relatively high following mild traumatic brain injury (mTBI).
- While boys sustain more mTBI, CRS in our sample was more prevalent among girls.
- African Americans/Blacks relative to Whites were more predisposed to CRS, an implication of risk-adapted intervention in reducing mTBI and CRS among children.

## Background

Approximately 1.6 to 3.8 million traumatic brain injuries (TBIs) occur each year in the USA [1–4]. The majority of these are concussions, a form of mild TBI (mTBI). It is estimated that 300,000 of these concussions are sports-related [5]. Since the 1990s, sports concussions have become a major health concern and public health challenge. Evidence of the long-term consequences of concussion has fueled efforts to improve its management. For example, De Beaumont and colleagues recently found that even retired athletes who sustained a concussion as long as 30 years prior to the study had significant cognitive and motor system alterations [6]. This underscores the concern for head injury in sporting activities. Due to the lack of widespread injury surveillance, however, many sports-related concussions go unrecognized. Youth athletes are at increased risk due to maximal brain plasticity during development [7]. Furthermore, Gessel et al. found that concussion accounts for more high school athletic injuries than collegiate athletic injuries [8]. In a study conducted by Marar and colleagues, concussion accounted for 13.2 % of all high school athletic injuries [9].

In addition to observed age differences in the incidence of sports-related concussions, a few studies have reported differences by gender and race/ethnicity. While male athletes have a higher concussion incidence overall, concussion incidence is higher among females for gender-comparable sports [1, 8–10]. Similarly, a large cohort (*n* = 1425) study on sex differences in outcome after mTBI observed significant odds of poorer outcomes following mTBI in girls relative to boys based on the post-concussion symptoms (PCS) score. In this cohort, 3 months after injury, males relative to females had a significant 38 % decreased odds of higher PCS scores, OR, 0.62, *p* < 0.001 [11].

Some studies have identified racial/ethnic differences in concussion incidence, awareness, outcome, and mortality. For example, Bloodgood et al. found that Blacks/African Americans and Hispanics were less likely to be aware of concussions than non-Hispanic Whites [12]. Langlois et al. indicated that death and hospitalization rates for Black/African American children 0–4 years old who sustained TBI were twice those of Whites [5]. Identifying and addressing these differences is essential in designing and implementing intervention efforts to reduce the incidence and complications of concussion among various groups.

One potential consequence of concussion is cognitive impairment, which affects memory, attention, and orientation. Outcomes of concussion in children could be devastating later in life with observed deficits in intellectual functioning and language acquisition. A study by Catroppa et al. assessed adaptive functioning 10 years post-injury in children with early TBI and observed poorer adaptive skills for those with more severe injury and behavioral difficulties [13]. Of the behavioral outcomes reported are difficulties with psychosocial functioning and psychiatric problems later in life [14, 15].

Impairment is often measured using one or more standardized test, including the Immediate Post-Concussion Assessment Cognitive Test (ImPACT). Along with sex and racial/ethnic disparities in head injury incidence and outcome, studies have also found evidence of disparities in levels of cognitive impairment after concussion within these groups. For example, results of several studies conducted by Covassin and colleagues revealed that female high school and collegiate athletes exhibited greater impairment in visual memory and more symptoms overall compared with males after concussion [1, 16]. A similar study conducted by Kontos and colleagues found differences in ImPACT composite scores by race, with Blacks/African Americans performing worse than Whites after concussion [17]. These outcome differences may be attributable to socioeconomic status and environmental factors, as well as biologic factors inherent within given racial/ethnic groups. A study on the predictors of neuropsychological test scores following mild TBI included race, education, history of learning problems, and days from injury to rehabilitation admission as the significant predictors of cognitive function. Relative to Whites, African Americans which constituted 36.8 % of the total study sample scored lower on all seven neuropsychological tests used for the assessment of cognitive function. The lower scores by African Americans were attributed to unequal access to educational opportunities and cultural differences as well as lack of equivalence in cognitive measures [11].

There remain unidentified factors related to the impact of mTBI and repetitive head trauma among children. These factors include though are not limited to social and cultural differences in predisposition to mTBI. Additionally, very little focus had been on mTBI and the impact of race and ethnicity among other health disparities indicators. Specifically, there are limited consistent data illustrating differences in concussion incidence, prevalence, and severity between boys and girls in sports. A few studies have examined the role of race, ethnicity, and socioeconomic factors in cognitive impairment immediately following concussion in children. Most available data only explore concussions in high school or collegiate athletes, whereas data are limited for children under the age of 13. The aims of the current study were to (1) characterize sports-related concussion among children, (2) assess the prevalence of cognitive-related symptoms following concussion, and (3) examine the relationship between CRS and race, sex as well as insurance.

## Methods

We conducted a retrospective study to examine sports-related concussion in relation to cognitive-related symptoms among children, following institutional review board (IRB) approval. To assess the response or outcome variable, namely cognitive-related symptom, in relation to health disparities indicators, a retrospective cohort design (case-only) was used. This design is effective, in spite of its retrospective nature, in obtaining evidence on the assessed relationship with risk ratio effect size. Using preexisting data from our electronic medical records, we examined all cases with concussion as our cohort.

### Study Population

The study population comprised children aged 2–19 years, who were diagnosed with sports-related concussion between January 2007 and June 2014. This consecutive sample consisted of all children regardless of race, ethnicity, and sex who met our eligibility criteria. To be included in this sample, participants were (a) diagnosed with concussion or TBI between 2007 and 2014, (b) children 2–19 years of age, (c) residing in DE Valley, and (d) underwent SCAT evaluation. We utilized consecutive sampling technique, thus including all children with sports-related concussion during the study period, between 2007 and mid-2014. Following the eligibility criteria, there were *n* = 1429 in our sample, with racial distribution indicating the following: White 1146 (80.2 %), Black/African American 170 (11.9 %), and other 113 (7.9 %).

### Study Variables

Concussion which is derived from Latin “concutere” refers to violent shaking of the brain, and represents the most common and less severe or mild traumatic brain injury (mTBI). Concussion reflects the disturbance in brain function as a result of direct or indirect force to the head. A direct impact to the brain is not required for concussion to occur, given the nature of this condition to be functional rather than structural injury due to shear stress to brain tissue as an outcome of rotational or angular forces [18]. The impact from concussion may result in bruising, blood vessels damage, and injury to the nerves. The third International Conference on Concussion in sports characterized concussion as a complex pathophysiologic process affecting the brain, induced by traumatic biomechanical forces [18].

#### Sport-Related Concussion Ascertainment

Concussion as related above is mild traumatic brain injury and could be characterized as invisible injury due to rapid acceleration or deceleration of brain tissues within the skull. These impacts may result in changes in the shape of the brain, brain stretch as well as brain cell damage. Additionally, the impulsive force may be transmitted beyond the head involving the face, neck, or elsewhere. Participants were involved in organized sports at school (football, baseball, softball, basketball, cheerleading, gymnastics, soccer) and were assessed using the symptom checklist. Girls who played soccer, softball, and basketball while boys who played football presented with majority of mTBI. To assist in the diagnosis of concussion, we used many tools to increase the sensitivity and specificity of the ascertainment. Specifically, we applied the post-concussion symptom scale, and concussion symptom inventory (CSI). Of these two instruments, we based our ascertainment on CSI since this is an empirically derived symptom checklist. Additionally, we used sideline assessment tools mainly Sport Concussion Assessment Tool (SCAT2) since it combines multiple assessment tools as well as physical examination to determine the possibility of concussion as mTBI. Therefore, our final assessment was based on SCAT2, the source for our data on CRS.

Children as patients provided the information during history taking and physical examination clinical encounter. However, for the every young ones, parents and caregivers serve as proxies to the children as respondents. The mean duration between injury and assessment (rehabilitation admission) at our facility was 7.6 days, SD 4.3.

Other variables studied included cognitive-related symptom, race, ethnicity, sex, age, insurance coverage, and length of hospitalization, as well as other variables that were available but not included in the final analysis, namely language, comorbidity, means of hospital arrival, and year of concussion.

#### Cognitive-Related Symptom (CRS) Ascertainment

Cognitive-related symptom was available as a string variable and was characterized as memory, orientation, and attention deficit. Specifically, we grouped symptoms and manifestation based on the variables in the medical records that reflected cognitive-related symptoms including a feeling of mental fogginess, problems concentrating, problems remembering, visual disturbance, amnesia, dizziness, a feeling of being slowed down, a feeling of increased emotion, and balance problems. We extracted these variables and used a binary scale to describe cognitive-related symptoms (0 = absence, 1 = presence). Cognitive-related symptom, based on our approach, indicated several attributes for a given case indicative of magnitude or degree of symptoms.

#### Other Variables Ascertainment (Race, Sex, Insurance, Age at Injury)

Race and ethnicity were self-reported. We collected data on those who self-identified as White, Black/African American, and some other race. Additional races represented in our data consisted of American Indian/Alaska Native, Asian, and Hawaiian Native/Other Pacific Islander. Similarly, participants self-identified as non-Hispanic/Latino or Hispanic/Latino. Self-reported variables have been utilized and deemed to be reliable with at least an estimated 80 % confidence [19]. Sex was available as male and female and was measured on a nominal scale. As recommended by the American Medical Association, we used boys and girls to represent the sex of our patients [20]. Furthermore, sex was critical to stratify sports-related concussion and was included in prevalence risk ratio test and adjusted risk ratio test. We examined insurance as both a proxy for income and access to available care following concussion. The insurance variable was classified into commercial (private), public (Medicaid), and uninsured/self-pay. We assessed the effect of insurance on cognitive-related symptoms using private/commercial insurance as the reference group. Data from our medical records were available for age on a continuous scale. To assess the effect of age on CRS as well as concussion, we utilized age as a continuous variable and transformed the age variable into categories, namely 2–9 years, 10–14 years, and 15–19 years. These ages at injury were treated as continuous variables which were later categorized to assess the impact of age on concussion as well as cognitive-related symptom.

### Statistical Analysis

The categorical and nominal variables including the prevalence of CRS were summarized using frequency and percentages, while the continuous scale variable was summarized using median and interquartile range (IQR) depending on the normality assumption. The chi-squared statistic, Pearson, and where necessary Fisher’s exact were used to characterize the categorical and nominal variables with concussion and cognitive-related symptoms. To examine the association between race, ethnicity sex, insurance, age, and age group with CRS, univariable and multivariable log binomial regression models were used. We built a multivariable log binomial regression model to examine the simultaneous effect of race, sex, insurance, age, and age group on CRS. We used forward loading and backward elimination and included all variables that were significant at 0.25 type I error, potential confounding variables with 10 % difference between the crude and stratified association and those that had a biologic or clinical relevance, such as age and sex. The significance level for the univariable model was 0.05 (95 % confidence interval), while the significance level for the multivariable model was 0.01 (99 % confidence interval) as to minimize type I error that may result from multiple comparison in the multivariable log binomial regression model. All tests were two-tailed, and the entire analyses were performed using Stata version 13.0 (Stata Corporation, College Station, Texas) [21].

## Results

Although not shown in the tables, 78.1 % of the children indicated measureable CRS. The prevalence of concussion was greater among boys (59.3 %) relative to girls (40.7 %). Table 1 presents the study characteristics of children with sports-related concussion stratified by race. There was a statistically significant sex distribution of children who sustained concussion by race. Among Blacks/African Americans, concussion was relatively higher among boys (71.8 %) compared to girls (28.2 %), whereas among Whites, concussion was marginally higher in boys (59.3 %) compared to girls (40.7 %), *χ*^2^(2) = 9.66, *p* = 0.008. A statistically significant difference by race was not observed in CRS, *χ*^2^(2) = 0.43, *p* = 0.81. Insurance coverage (implying access to care) showed a statistically significant variation by race. Whites were more likely to have private/commercial insurance relative to Blacks/African Americans (Whites 90.3 %, Blacks/African Americans 63.5 %), *χ*^2^(4) = 105.1, *p* < 0.0001.

**Table 1:** Study characteristics of children with sports-related concussion, stratified by race

Variable	Race				
	White	Black/African American	Some Other Race	*χ* ^2^(*df*)	*p*
	*n* (%)	*n* (%)	*n* (%)		
Sex				9.66 (2)	0.008
Male	680 (59.3)	122 (71.8)	70 (62.0)		
Female	466 (40.7)	48 (28.2)	43 (38.0)		
Cognitive-related Symptoms				0.43 (2)	0.81
Yes	899 (78.4)	130 (76.5)	87 (76.3)		
No	247 (21.6)	40 (23.5)	27 (23.7)		
Insurance Coverage				105.1 (4)	<0.0001
Private	1035 (90.3)	108 (63.5)	83 (73.5)		
Public	67 (5.9)	43 (25.3)	19 (16.8)		
Uninsured	44 (3.8)	19 (11.2)	11 (9.7)		
Language				0.34 (2)	0.84
English	834 (72.8)	123 (72.4)	85 (75.2)		
Spanish	312 (27.2)	47 (27.6)	28 (24.8)		
Year Injured				36.2 (14)	0.001
2007	6 (0.5)	0 (0.0)	0 (0.0)		
2008	33 (2.9)	3 (1.8)	2 (1.8)		
2009	134 (11.7)	19 (11.2)	2 (1.8)		
2010	182 (15.9)	38 (22.3)	11 (9.7)		
2011	205 (17.9)	24 (14.1)	18 (15.9)		
2012	163 (14.2)	26 (15.3)	24 (21.2)		
2013	293 (25.6)	50 (29.4)	34 (30.1)		
2014	130 (11.3)	10 (5.9)	22 (19.47)		

Notes and abbreviations: The type 1 error tolerance (significance level) was set at 5 % (0.05). *n* = number, % = percentage, *df* = degree of freedom, Pearson Chi-squared = *χ* ^2^

The characterization of the study variables by ethnicity is illustrated in Table 2. Although there was no significant difference in the distribution of sex by ethnicity among children with concussion, non-Hispanic/Latino girls were less likely to have concussion relative to their Hispanic/Latino counterparts. CRS, although statistically insignificant, was more common among Hispanic/Latino children (81.4 %) relative to non-Hispanic/Latino children (77.8 %) *χ*^2^(1) = 0.69, *p* = 0.41. Similar to insurance coverage in the racial distribution, a significant ethnic variation was observed, with non-Hispanic/Latino children more likely to have private insurance compared to Hispanic/Latino children (non-Hispanic/Latino 86.7 %, Hispanic/Latino 73.5 %), *χ*^2^(2) = 14.6, *p* = 0.001.

**Table 2:** Study characteristics of children with sports-related concussion, stratified by ethnicity

Variable	Ethnicity			
	Non-Hispanic/Latino	Hispanic/Latino	*χ* ^2^(*df*)	*p*
	*n* (%)	*n* (%)		
Sex			0.80 (1)	0.37
Male	814 (61.3)	58 (56.9)		
Female	513 (38.7)	44 (43.1)		
Cognitive-related Symptoms			0.69 (1)	0.41
Yes	1033 (77.8)	83 (81.4)		
No	294 (22.2)	19 (18.6)		
Insurance Coverage			14.6 (2)	0.001
Private	1151 (86.7)	75 (73.5)		
Public	110 (8.3)	19 (18.6)		
Uninsured	66 (5.0)	8 (7.8)		
Year Injured			21.3 (7)	0.003
2007	6 (0.5)	0 (0.0)		
2008	37 (2.8)	1 (1.0)		
2009	149 (11.2)	6 (5.9)		
2010	223 (16.8)	8 (7.8)		
2011	233 (17.6)	14 (13.7)		
2012	197 (14.8)	16 (15.7)		
2013	341 (25.7)	36 (35.3)		
2014	141 (10.6)	21 (20.6)		

Notes and abbreviations: The type 1 error tolerance (significance level) was set at 5 % (0.05). *n* = number, % = percentage, *df* = degree of freedom, Pearson Chi-squared = *χ* ^2^

The crude association between CRS and the independent variables race and sex, as well as other study variables is demonstrated in Table 3. A significant racial variation was not observed with respect to CRS, *p* > 0.05. Girls were more likely to have CRS following sports-related concussion, with an observed 7 % increased likelihood compared to boys, risk ratio (RR) = 1.07, 95 % CI 1.01–1.13, *p* = 0.02. Although statistically insignificant, compared to children with private insurance, the uninsured children were 6 % more likely to have cognitive impairment following sports-related concussion, RR = 1.06, 95 % CI 0.95–1.18, *p* = 0.31. A significant association was observed between age and CRS, with older children presenting with greater risk. Compared to children 2–9 years, children 10–14 years had 13 % increased risk of sustaining CRS following concussion (RR = 1.13, 95 % CI 1.00–1.28, *p* = 0.06) and children 15–19 years had a 19 % increased risk, RR = 1.19, 95 % CI 1.05–1.35, *p* = 0.005.

**Table 3:** Univariable log binomial regression model of the effect of race and other factors on cognitive-related symptoms

Variable	Prevalence Risk Ratio (RR)	95 % CI	*p*
Race			
White	1.0	Referent	Referent
Black/African American	0.97	0.89–1.07	0.57
Some Other Race	0.98	0.88–1.09	0.73
Sex			
Male	1.0	Referent	Referent
Female	1.07	1.01–1.13	0.02
Insurance Coverage			
Private	1.0	Referent	Referent
Public	1.0	0.90–1.10	0.92
Uninsured	1.06	0.95–1.18	0.31
Age Group			
2–9	1.0	Referent	Referent
10–14	1.13	1.00–1.28	0.06
15–19	1.19	1.05–1.35	0.005
Language			
English	1.0	Referent	Referent
Spanish	0.96	0.90–1.03	0.25

Notes and abbreviations: The type 1 error tolerance (significance level) was set at 5 % (0.05). *n* = number, % = percentage

The simultaneously adjusted risk model is shown in Table 4. After controlling for insurance, age, sex, and length of hospitalization, Blacks/African Americans had a significant 77 % higher risk of CRS after concussion relative to Whites, adjusted prevalence risk ratio (aPRR) = 1.77, 99 % CI, 1.02–3.08.

**Table 4:** Multivariable log binomial regression model for the effect of race/ethnicity on cognitive-related symptoms

Variable	Adjusted Risk Ratio	99 % CI	*p*
Race			
White	1.0	Referent	Referent
Black/African American	1.77	1.02–3.08	0.008
Some other race	0.7	0.10–4.93	0.64
Sex			
Male	1.0	Referent	Referent
Female	0.79	0.39–2.04	0.53
Insurance coverage			
Private	1.0	Referent	Referent
Public	0.72	0.30–1.71	0.33
Age group			
2–9	1.0	Referent	Referent
10–14	3.62	0.66–19.86	0.052
15–19	5.61	0.48–65.26	0.07
Length of stay (days)	1.01	0.99–1.02	0.19

Notes and abbreviations: The type 1 error tolerance (significance level) was set at 1 % (0.01). *n* = number, % = percentage, *df* = degree of freedom, Pearson Chi-squared = *χ* ^2^

## Discussion

This study was performed to characterize concussion sustained among children who play sports and were seen in our pediatric institution. We postulated that patients who sustained concussion present with substantial CRS and that differences in outcome may be observed by race, ethnicity, and other health disparities indicators. Using an explanatory multivariable binomial regression (predictive) model, we obtained some relevant findings. (1) The occurrence of CRS is relatively high among children who play sports and sustained concussion. (2) CRS is associated with sex, with more girls reporting impairment relative to boys. (3) There is a substantial relationship between age and CRS. (4) After controlling for potential confounding, such as insurance, age, sex, and length of hospitalization, Black/African American children were more likely to develop CRS following sports-related concussion.

Racial variances in CRS were not observed in our crude (unadjusted) model. This observation may be due to the use of a binary variable in the measurement of CRS. In our data, we observed several indicators of CRS that required the use of ordinal scale to rank this variable. Therefore, the application of ordered category might result in the observation of a significant difference in CRS by race. We built a multivariable model using log binomial regression for possible explanation, adjusting for biologic and known confounding factors for racial association with disease and health conditions. The multivariable explanatory model clearly indicated racial variance in CRS following sports-related concussion. These data consequently support a previous studies in comparable settings. While a prospective case-control study did not observe a significant difference between African and Whites on baseline or post-concussion verbal memory, visual memory, reaction time, and total reported symptoms, however, African Americans were almost 2.5 times as likely to have at least one clinically significant cognitive decline on ImPACT at 7 days post-concussion, as well as scored lower at 7 days post-concussion relative to baseline on processing speed [17]. In another study, relative to Whites, African Americans that constituted 36.8 % of the total study sample scored lower on all seven neuropsychological tests (verbal memory—total and delayed, working memory—WAIS-3 DS and WAIS 3 LNS, perceptual reasoning, visual memory, processing speed) used for the assessment of cognitive function. These racial variances were attributed by authors attributed to unequal access to educational opportunities and cultural differences as well as lack of equivalence in cognitive measures [22].

The observed racial variation in CRS following concussion may be due to the delay in diagnosing and treating concussion, which correlates with poor prognosis. Our finding is supported by a few studies in prior research [17] that examined post-concussion ImPact composite score. The differences in these scores with Blacks/AA performing poorer relative to Whites may be attributed to several factors including baseline variance, stop socioeconomic status that may impact on timely access to care. *While concussion has been shown in literature to vary in prevalence and incidence by race*, *its sequelae has not been fully described using health disparities indicators. Our study therefore represents a preliminary finding in this vein*.

Whereas sports-related concussion has been previously described with respect to sex, however, this study described the prevalence of CRS following mTBI by sex. We have demonstrated that, following concussion, girls have increased risk of sustaining cognitive impairment. While this finding is interesting, we are unable to provide any biologic or psychosocial explanation to justify our observation. However, it is plausible to suggest that it may require longer duration for girls to be seen if sports activities are not very well developed in their institutions, implying lack of resources and timely intervention. Despite these difficulties in arriving at a specific explanation, studies have indicated the magnitude and severity of concussion among males relative to females [23]. Differences are suggested to be attributable to differences in head mass and acceleration, as well as other biologic determinants of altered mentation and visual acuity [24]. Furthermore, the increased prevalence in CRS among girls may be attributable to their increasing willingness to disclose their symptoms compared to boys. At this particular competitive level, boys may not like to disclose their symptoms, since doing so may limit their participation in sports [16]. Additionally, a study on sex differences in outcome after mTBI observed significant odds of poorer outcomes following mTBI in girls relative to boys based on the post-concussion symptoms (PCS) score [11]. In effect, we caution the interpretation of increased prevalence in CRS among girls without a careful assessment of other factors related to pain perception and expression.

Insurance has been reported in previous literature to facilitate access and utilization of care. Our studies showed that Blacks/African Americans were less likely to have private insurance compared to their White counterparts, aligning with previous findings illustrating poor outcomes for children with public insurance as well as those who are uninsured [25]. The observed association between insurance coverage and CRS in our sample may be due to decreased access to care as well as decreased care utilization by Blacks/African Americans.

The age at injury was shown in our data to significantly influence CRS. Indeed, we observed a significant association between age and CRS, with older children presenting more with CRS following concussion. The observed association may be due to participation in high-energy contact sports by older children. Willer et al. observed that older children were more likely to sustain head injuries and symptoms of concussion from sports activities, while primary cause of injury for young children was fall [26]. These sports are available to older children and are conducive to an increase in the degree of trauma, which in turn compromises cognitive functions, such as attention, memory, and orientation. It is also worthwhile to consider the changing plasticity of the brain as the child grows, implying the greater ability of the young brain to adjust to change during injury compared to older children with increased vulnerability to mTBI. McKinlay and Anderson examined age at injury as one of the issues associated with preschool child traumatic brain injury. Firstly, this study acknowledged the communication difficulties that may complicate deficits assessment in younger children. Secondly, that the absence of deficit should be cautiously interpreted since the problem or deficit may become apparent over time as the age-dependent skills fail to emerge [27, 28]. Subsequently, our data the association between age at injury and mTBI supports the observation that neuroplasticity should not be interpreted as the capability of the young child brain to return to pre-injury function [27].

This study is not without limitations. (1) This is a retrospective design that may be influenced by information and selection biases, as well as the lack of temporality, implying cause and effect association. (2) The use of preexisting data from our electronic medical records precluded data collection of known variables that could provide further explanation regarding the relationship between cognitive impairment and race. As a result of this limitation, it is possible to suspect unmeasured confounding to influence the result in this study. (3) In addition, like in most epidemiologic studies, residual confounding may influence the findings in this sample, since no matter how sophisticated a model used for adjusting for confounding, residual confounding persists [29].

## Conclusions

In summary, there are racial, age, and sex disparities with respect to CRS following sports-related concussion in children. However, due to the retrospective nature of our design, caution should be exercised in the interpretation and application of these findings to public health interventions focused on minimizing CRS following sports-related concussions.
